# Supplementary material for: A High-Linearity Glucose Sensor Based on Silver-Doped Con A Hydrogel and Laser Direct Writing
Source: Polymers (Basel). 2023 Mar 13;15(6):1423. doi: 10.3390/polym15061423 (PMC10053202; doi:10.3390/polym15061423)
Supplement: Supplementary file 1 [file polymers-15-01423-s001.zip › polymers-2218296-supplementary.pdf]

Supporting Information

A high-linearity glucose sensor based on silver-doped Con A hydrogel and laser direct writing

Yulin Hu [1,#], Dasheng Yang [1,#], Hongbo Zhang [1], Yang Gao [1,2,*], Wenjun Zhang [3], Ruixue Yin [1,*]

1 School of Mechanical and Power Engineering, East China University of Science and Technology, 130 Meilong Road, Shanghai, 200237, China.

2 Shanghai Key Laboratory of Intelligent Sensing and Detection Technology, East China University of Science and Technology, 130 Meilong Road, Shanghai, 200237, China.

3 Division of Biomedical Engineering, University of Saskatchewan, 57 Campus Drive, Saskatoon, SK S7N 5A9, Canada

* Correspondence: yanggao@ecust.edu.cn; yinruixue@ecust.edu.cn

# These authors contributed equally to this work.

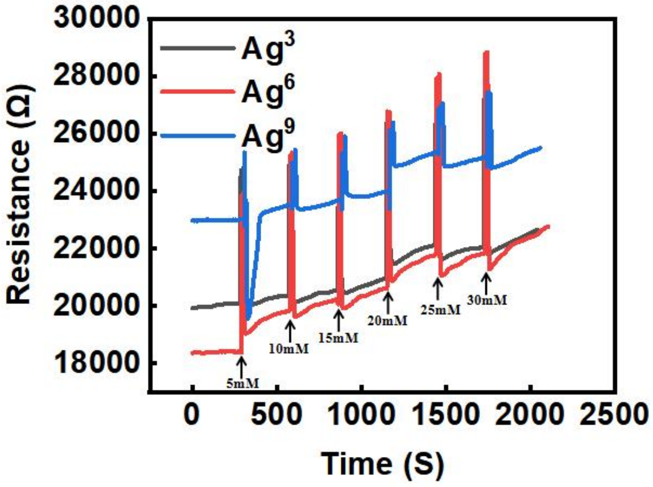

Fig. S1. Time-resolved capacitance in response to continuous glucose concentration changes range from 0-30 mM.
